# Intelligent treatment strategies for knee cartilage injury repair

**DOI:** 10.3389/fsurg.2026.1763744

**Published:** 2026-03-26

**Authors:** Sheng Li, Wei Wang, Liang A

**Affiliations:** 1 Department of Orthopaedics, Central Hospital Affiliated to Shenyang Medical College, Shenyang, Liaoning, China; 2 Shenyang Hand Foot Clinical Research Center, Shenyang, Liaoning, China; 3 Hainan Vocational University of Science and Technology, Haikou, Hainan, China

**Keywords:** cartilage repair, intelligent surgery, knee cartilage injury, personalized medicine, surgical navigation, tele-rehabilitation

## Abstract

Knee cartilage injury, a prevalent clinical condition leading to pain and functional impairment, presents significant challenges for complete restoration due to the tissue's limited regenerative capacity. This narrative review examines the paradigm shift towards intelligent treatment strategies encompassing the entire patient journey, from preoperative planning to postoperative rehabilitation. We detail the application of advanced surgical navigation systems (e.g., Holoknee), robotic assistance, and artificial intelligence (AI) in enhancing the precision of diagnostic assessment, surgical execution, and personalized planning based on the International Cartilage Repair Society (ICRS) classification. Furthermore, we evaluate the role of intelligent rehabilitation equipment, wearable sensors, and telemonitoring systems in facilitating real-time feedback, improving adherence, and optimizing functional recovery remotely. The synthesis of evidence indicates that integrating intelligent technologies with established surgical techniques—such as microfracture, autologous chondrocyte implantation (ACI), matrix-assisted chondrocyte implantation (MACI), and mesenchymal stem cell (MSC) therapies—can significantly improve surgical accuracy, reduce complications, and streamline rehabilitation. This review concludes that the future of knee cartilage repair lies in a holistic, data-driven, and personalized approach, where intelligent systems bridge the gap between innovative surgical repair and effective long-term functional restoration, although further standardization and clinical validation are required.

## Graphical Abstract

**Figure:**
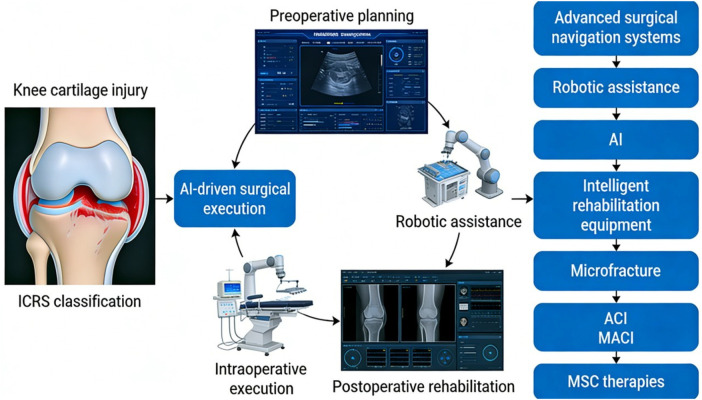

## 1 Introduction

Knee cartilage injury is a major cause of joint pain, functional disability, and a primary precursor to osteoarthritis (OA). The avascular and aneural nature of articular cartilage severely limits its intrinsic healing potential, making effective and durable repair one of the most formidable challenges in orthopaedic surgery (1). Traditional treatment algorithms rely heavily on surgeon experience and standardized protocols for techniques such as microfracture, osteochondral grafting, and autologous chondrocyte implantation (ACI). While these methods have demonstrated efficacy, outcomes can be inconsistent, influenced by factors including lesion size, location, patient biology, and the precision of surgical execution (2).

The advent of the digital health revolution has ushered in a new era of “intelligent orthopaedics,” characterized by the integration of advanced technologies to augment every phase of patient care. In preoperative planning, artificial intelligence (AI) and three-dimensional (3D) modeling enable precise lesion characterization and simulation of surgical outcomes. Intraoperatively, computer navigation and robotic-assisted systems, such as the Holoknee platform, promise sub-millimeter accuracy in instrument guidance and implant placement, potentially surpassing the limits of conventional manual techniques (3, 4). Postoperatively, the landscape is being transformed by intelligent rehabilitation devices and remote monitoring systems. These technologies, leveraging sensors, augmented reality (AR), and telemedicine, allow for objective assessment of rehabilitation metrics, personalized exercise modulation, and continuous patient-provider communication outside the clinical setting (5).

While previous reviews have comprehensively examined individual components of intelligent orthopaedics—In the realm of surgical navigation, such as meta-analyses by Luan et al. (3) and Lei et al. (4) demonstrated that computer navigation and robotic assistance in total knee arthroplasty significantly improve component alignment and reduce outliers compared to conventional instruments, though postoperative clinical outcomes showed no significant differences. Moglia et al. (6) explored the application of mixed reality and artificial intelligence in knee osteotomy, finding that the Holoknee system enhanced surgical training and preoperative planning efficiency—a comprehensive synthesis of how these technologies interlink to form a cohesive treatment continuum, from AI-enhanced diagnosis through robot-aided surgery to data-driven rehabilitation, is lacking. This integrated perspective is essential because the true potential of intelligent technologies lies in their synergistic application across the entire care pathway, where preoperative planning data can inform intraoperative execution, and postoperative recovery metrics can refine future surgical algorithms. This review aims to fill this gap. This review aims to critically examine: (1) the application and evidence for intelligent surgical systems (navigation and robotics) in improving repair accuracy; (2) the role of intelligent rehabilitation and remote monitoring in enhancing recovery outcomes and adherence; and (3) how these technologies synergize with established surgical techniques to facilitate truly personalized treatment planning based on individual patient and lesion profiles. By providing this integrated perspective, we highlight the transformative potential of intelligent strategies to improve the precision, efficacy, and accessibility of knee cartilage injury repair.

## 2 Methods

This article is a narrative review designed to provide a comprehensive overview of intelligent treatment strategies for knee cartilage injury repair. A literature search was conducted using PubMed, Scopus, and Web of Science on December 9, 2025, with the following keywords: “Knee Cartilage Injury,” “Intelligent Surgery,” “Surgical Navigation,” “Tele-Rehabilitation,” “Personalized Medicine,” and “Cartilage Repair.” Boolean operators (AND, OR) were applied to combine search terms appropriately.

Given the narrative format, no formal systematic review protocol was registered, and a PICOS framework was not applied. However, to ensure transparency and reproducibility of the literature base, the following criteria guided study selection: (1) peer-reviewed original research, systematic reviews, or meta-analyses; (2) studies addressing surgical navigation, robotic assistance, AI applications, or digital rehabilitation technologies in the context of knee cartilage pathology or related orthopaedic procedures; (3) English-language publications; (4) no date restrictions to capture the evolution of these technologies. Studies were selected based on relevance to the three thematic pillars of this review: intelligent surgical systems, intelligent rehabilitation, and personalized treatment planning. Given the expert-driven nature of this narrative synthesis, formal quality assessment tools (e.g., GRADE) were not applied, but the level of evidence is noted throughout the discussion where appropriate.3. Intelligent treatment strategies for knee cartilage injury repair.

### 2.1 Applying intelligent surgical robots and navigation systems in the repair of knee cartilage injuries

The Holoknee (6) is an advanced surgical navigation system that combines optical tracking, computer vision, and robotics to achieve high-accuracy guidance during knee surgery. The system involves several key aspects.

3D holographic image reconstruction: The Holoknee system first obtains 3D data of the knee joint using special imaging equipment. Microsoft Hololens 2 provides the greatest field of view (FOV52°) and the wider display spatial resolution of 2,048 × 1,080, Binocular, Microphone Presence, Camera:1080p, Operating System:W-Holo. These data are processed using a computer to produce an accurate 3D holographic image that can display the internal structure of the knee joint in real time, including the bone, cartilage, and ligaments.

Optical tracking and positioning: The system uses an optical tracker to track the position and orientation of the instrument in real-time. These trackers ensure the accurate navigation of surgical instruments by emitting and receiving infrared light signals for real-time communication with a computer.

Computer navigation and planning: Based on 3D holographic images and optical tracking data, the computer system can generate detailed surgical planning, including the cutting path, implant position, and angle. This information is directly displayed above the patient's knee using a holographic projection technique, which provides the physician with intuitive surgical guidance.

Holoknee was developed by incorporating:

- A file-manager Python application to manage patient data organization remotely on Microsoft Azure cloud storage;
- Unity-based application, exploiting Mixed Reality Toolkit (MRTK), running on board the HoloLens 2;
- An interoperable client-server application based on OpenICE (Open-source Integrated Clinical Environment) protocol (https://www.openice.info/) for gathering vital signs from in-room medical equipment and delivery in real-time to the holographic visualization.

Robotic-assisted operation: In some cases, the Holoknee system can be combined with a robot-assisted system to achieve higher surgical accuracy. The robot can accurately perform operations such as cutting, grinding, and implantation according to the computer system instructions, to reduce human errors.

**Figure 1:**
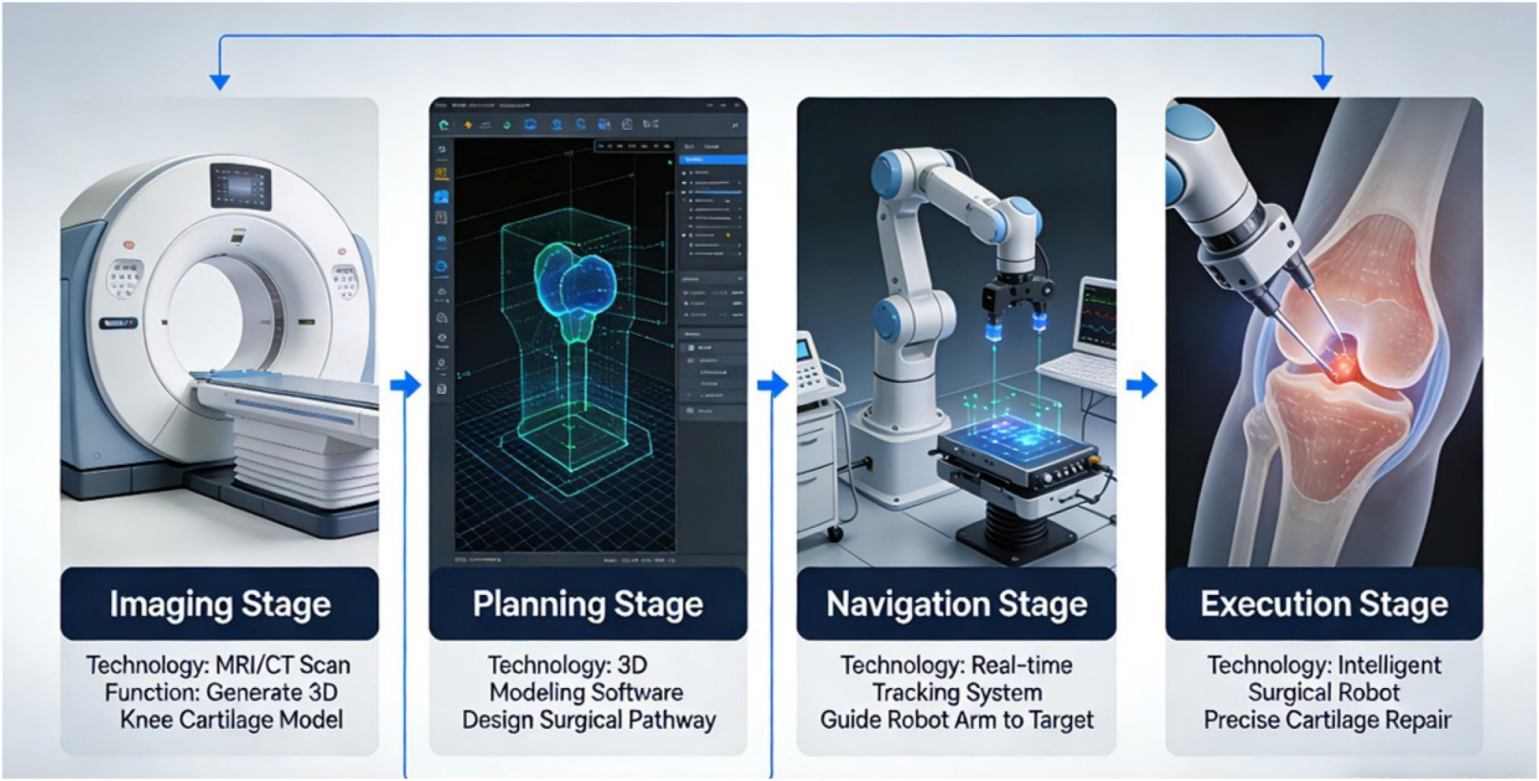
Overview of the application of intelligent surgical robots and navigation systems in the repair of knee cartilage injuries.

A meta-analysis compared the use of conventional instruments (CONI), accelerometer-based navigation (ABN), and computerised navigation (CN) systems in total knee arthroplasty (TKA). The ABN system provides more precise component alignment and fewer outliers than CONI. In addition, the ABN system requires a shorter procedural time than the CN system. However, the postoperative clinical outcomes (PCO) among the three systems are not significantly different. Another network meta-analysis compared different techniques used in TKA, including computer navigation, patient-specific instruments, surgical robotics, and conventional instruments and found that navigation and robotics reduced misalignment compared to patient-specific instruments and conventional instruments. Navigation also results in higher mid- and long-term KSS knee scores than conventional instruments. It is important to acknowledge that the strongest evidence for navigation and robotic systems in knee surgery derives from total knee arthroplasty (TKA) literature (3, 4). While these studies demonstrate improved alignment accuracy and reduced outliers with navigation systems, their direct applicability to cartilage repair procedures requires careful consideration. Cartilage repair presents distinct technical challenges compared to TKA: precise debridement of the lesion borders, management of the calcified cartilage layer without damaging subchondral bone, and accurate graft implantation or fixation. The precision gains demonstrated for bone resections and implant alignment in TKA may translate to improved accuracy in these cartilage-specific tasks, but this remains extrapolation rather than direct evidence. Future studies specifically evaluating navigation and robotic assistance for cartilage repair procedures (microfracture, ACI/MACI, OAT) are needed to validate this translational assumption. One study combined AI and hybrid reality (MR) for surgical planning of knee osteotomy; participants performed the task faster in the second trial than in the first, suggesting that MR can be used effectively for surgical training and preoperative planning. The study concluded that the Holoknee positively impacted surgical training, preoperative planning, and intraoperative guidance for knee osteotomies (6). In summary, intelligent surgical robots and navigation systems have shown potential for improving the accuracy and efficiency of knee cartilage injury repair surgeries (see Figure 1).

### 2.2 Applying intelligent rehabilitation equipment and remote monitoring systems in postoperative rehabilitation of knee cartilage injury repair

Oliva et al. provide this framework specifically for orthopaedic surgery—discussing the advantages, limitations, and current state of telemonitoring systems in musculoskeletal care (7). Intelligent rehabilitation devices can accurately monitor and evaluate a patient's motor status, muscle strength, and joint range of motion (ROM) by applying advanced technologies such as robotic technology, sensors, and AI. These devices can provide patients with personalised rehabilitation programs and adjust treatment programs in real time according to the rehabilitation progress, thereby improving rehabilitation outcomes. For example, intelligent rehabilitation devices can effectively prevent deep venous thrombosis by simulating the muscle activity of healthy individuals and improving the haemodynamics of patients.

Research has shown that a new, economically feasible rehabilitation training monitoring and evaluation system enables real-time assessment of exercises and provides precise insights into deviations from correct execution. The assessment consists of two important components: ROM classification and compensatory pattern recognition. The mean accuracy of the assessed ROM categories was 89% (95% CI: 84%–94%), and the mean accuracy of the categorical compensation mode was 98% (95% CI: 96%–100%), based on a validation study of 45 patients performing 1,080 rehabilitation exercises (8). This cutting-edge system significantly improves rehabilitation practices by complementing traditional rehabilitation assessments performed by skilled clinicians. Additionally, its combination with home rehabilitation programs can greatly improve the therapeutic effect on patients and increase the chances of obtaining high-quality care (8). Rehabilitation systems based on AR have also been explored, and they may be useful alternatives to conventional rehabilitation (9).

Remote monitoring systems can also facilitate communication between physicians and patients, enabling real-time feedback and personalized guidance (10). Doctors can remotely monitor the progress of patient rehabilitation, detect problems in a timely manner, and provide guidance. Patients can obtain advice from doctors by systematically providing feedback on their rehabilitation, which greatly shortens the distance between doctors and patients and improves the efficiency and quality of rehabilitation. One study showed that these systems support the early rehabilitation of patients, with high rates of acceptance and adherence, and early results showed a significant improvement in the overall quality of life (*p* < 0.001, Cohen's *d* = 0.82), significant reduction in pain (*p* < 0.01, *d* = 0.64), and significant reduction in depression (*p* < 0.01, *d* = 0.58) (11). Telemonitoring systems are used to monitor the ROM, mobility, patient-reported outcome measures, and compliance with home exercise programs. Studies have shown that telemonitoring systems can significantly reduce the rate of rehospitalisation (12). The use of smartphone applications and wearable devices is particularly common (13); these technologies enable remote guidance and monitoring of the patient's progress and may replace face-to-face rehabilitation programs (14, 15). Patients often require prolonged immobilisation and rest after knee cartilage injury repair, which may lead to muscle atrophy and joint stiffness. Intelligent rehabilitation devices can assist patients with appropriate muscle exercises and joint movements and help prevent muscle atrophy and joint stiffness. In contrast, the remote monitoring system can ensure that patients receive timely guidance and help and reduce risks during rehabilitation. In addition, the application of intelligent rehabilitation equipment and remote monitoring systems can help doctors better manage pain in patients (16). By monitoring pain in real-time, physicians can adjust the treatment plan to provide more effective pain management.

Despite the promising results, several limitations of the current evidence base must be acknowledged. First, significant heterogeneity exists among technology platforms, with different studies evaluating different sensors, smartphone applications, and monitoring systems, making direct comparisons difficult. Second, outcome measures vary considerably across studies, ranging from objective metrics (ROM, step count) to patient-reported outcomes (pain, quality of life), with no standardized core outcome set for digital rehabilitation studies. Third, most studies report only short- to mid-term follow-up (3–12 months), leaving questions about long-term adherence and durability of benefits unanswered.

Furthermore, the implementation of intelligent rehabilitation faces practical challenges. The “digital divide” means that elderly patients, those with lower socioeconomic status, or those in rural areas with limited internet connectivity may not equally benefit from these technologies. Patient technological literacy varies widely, and inadequate training or support can lead to frustration and abandonment of digital tools. Data security and privacy concerns are paramount when transmitting health information through smartphone applications or cloud-based platforms, requiring robust encryption and compliance with regulations such as HIPAA or GDPR. Finally, reimbursement models for tele-rehabilitation services remain inconsistent across healthcare systems, potentially limiting widespread adoption despite clinical evidence of benefit. Addressing these challenges will be essential for equitable and sustainable implementation of intelligent rehabilitation.

**Figure 2:**
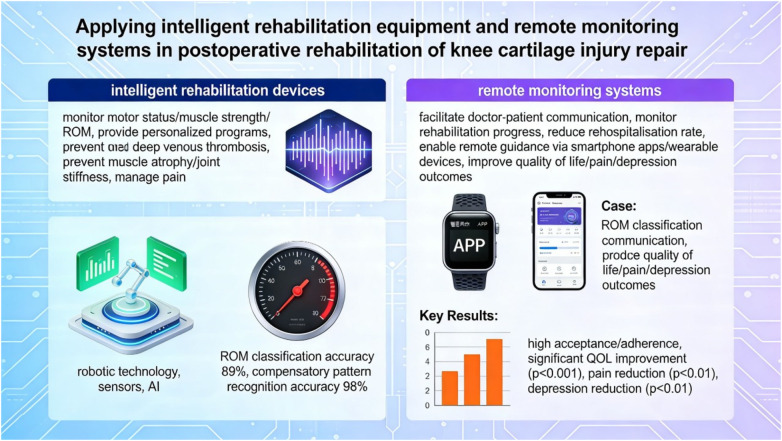
Overview of intelligent rehabilitation and remote monitoring systems for postoperative knee cartilage repair. This figure illustrates the components and workflow of intelligent rehabilitation, including wearable sensors for real-time motion tracking (accelerometers, gyroscopes), smartphone applications for patient guidance and data collection, and telemedicine platforms for remote clinician oversight. Key functions include: range of motion (ROM) assessment, compensatory movement pattern recognition, exercise adherence monitoring, and bidirectional patient-provider communication. The integration of these technologies creates a continuous feedback loop that personalizes rehabilitation protocols and enables early detection of complications. AI, artificial intelligence; ROM, range of motion; PROM, patient-reported outcome measures; SMS, short message service; App, application.

It should be noted that the studies cited in this section employed various commercially available and research-grade devices; however, the original publications often did not specify brand names or model numbers, limiting the ability to provide complete technical specifications. Future research should adhere to reporting guidelines that require detailed device characterization to enhance reproducibility (see Figure 2).

### 2.3 Intelligent personalized treatment for knee cartilage injury repair

#### 2.3.1 Development of individualized treatment plan

Preoperative planning: AI technology is used to perform 3D reconstructions and simulation knee surgeries, and personalised treatment is performed according to the size, depth, location, patient preference, and nature of the lesion (cartilage or osteochondral) according to the International Society for Cartilage Repair (ICRS) score (17) Table 1, to determine the optimal surgical plan and repair strategy (18). This allows physicians to fully understand and prepare for the procedure preoperatively, thereby increasing its success rate.

**Table 1:** Classification of knee cartilage injury repair based on the international cartilage repair society (ICRS) score.

Repair grade	Type	Clinical manifestation
Ⅰ	A	Repaired surface is intact and smooth
	B	Repaired surface is flush with surrounding cartilage
	C	Complete graft fusion with surrounding cartilage
II	A	Fibrillary surface after repair
	B	75% of cartilage damage depth repaired
	C	Graft has <1 mm gap with surrounding cartilage
III	A	Dispersed small cracks on repaired surface
	B	50% of cartilage damage depth repaired
	C	3/4 of graft fused with surrounding cartilage and >1 mm gap with the cartilage
IV	A	Several small or a few large cracks in repaired surface
	B	<25% of cartilage damage depth is repaired
	C	<1/2 of graft fused with surrounding cartilage and significantly >1 mm gap with the cartilage

The ICRS classification system provides a standardized framework for characterizing cartilage lesions, which directly informs treatment selection. Intelligent technologies enhance the utility of this classification in several ways. First, AI-powered 3D reconstruction from MRI or CT imaging enables more precise measurement of lesion dimensions and depth than conventional arthroscopic assessment alone, potentially reducing inter-observer variability in ICRS grading. Second, surgical navigation systems can intraoperatively verify that debridement extends to stable borders as defined by the ICRS grade, ensuring complete removal of damaged cartilage. Third, for grade III and IV lesions requiring graft implantation, navigation guidance can help achieve the “flush” interface with surrounding cartilage that defines successful repair in grade I outcomes. Thus, intelligent technologies not only assist in executing treatments appropriate to each ICRS grade but also improve the accuracy of the grading itself.

#### 2.3.2 The specific surgical methods are as follows

Microfracture is a bone marrow stimulation technique commonly used to treat smaller lesions localized to the cartilage. A systematic review (19) of microfracture for full-thickness articular cartilage defects (mean lesion size 3.4 ± 2.1 cm^2^) reported failure rates ranging from 11% to 27% at 5 years and 6% to 32% at 10 years, with the wide ranges reflecting heterogeneity in patient characteristics, lesion location, and definition of failure across the 28 included studies. Radiographic analysis of 10 studies reporting second-look arthroscopy (*n* = 205 patients) revealed good integration of the fibrocartilage repair tissue, confirming that microfracture has good function and an analgesic effect in the medium term and satisfactory clinical results (19). Autologous chondrocyte implantation (ACI) and modified ACI (ACI-C) or matrix-assisted costal chondrocyte implantation (MACI) are commonly used to treat larger simple osteochondral lesions. Clinical results of modified ACI (ACI-C or MACI) for the treatment of knee cartilage injuries have shown significantly improved prognoses of patients at the 5-year mid-term follow-up compared with those of microfracture (20). Autogenous osteochondral transplantation (OAT) is recommended for smaller osteochondral lesions, including subchondral injuries, whereas osteochondral allografts (OCA) are recommended for larger osteochondral lesions (21). Cartilage fragmentation implantation (MCI) is a one-step procedure that requires no separate cell processing and is a promising method for small defect cartilage repair (mean: 2.77 cm^2^, range: 1.3–4.7 cm^2^), with minced cartilage showing almost the same improvement in short-term clinical scores as standard cartilage repair techniques (IKDC, KOOS) (22). Cell-based therapies, especially those involving mesenchymal stem cells (MSCs) (23), have shown promise in regenerating cartilage and improving patient outcomes and are a safe one-stage surgical solution that provides short-term clinical improvement and satisfactory cartilage repair; one study used MSCs at a concentration range of 3.96–11.9 × 10^6^ total cells (24, 25). See Table 2.

**Table 2:** Treatment modalities and outcomes based on injured cartilage area (17).

Damaged area	Damage degree	Treatment mode	Outcome			Smart technology
<2.5 cm^2^	Low demand	Debridement	Fail	Subchondral bone damage	OAT, bone graft + ACI/MACI, TKA	Artificial intelligence planning, surgical navigation, robot assistance
	High demand	MFX, OAT, ACI/MACI, Hydrogel, MCI				
2.5–8 cm^2^	Low demand	Debridement				
	High demand	AMIC, OCA, OAT, ACI/MACI, Hydrogel, MCI, MSCs		No damage to subchondral bone	ACI/MACI, MCI, MSCs	
>8 cm^2^	Traumatic bone defects or bone loss	OCA, TKA				

Cartilage injury treatment modalities: MFX, microfracture; OAT, osteochondral autograft transfer; ACI, autologous chondrocyte implantation; OCA, osteochondral allografts; MCI, minced cartilage implantation; TKA, total knee arthroplasty; MACI, matrix-assisted chondrocyte implantation; AMIC, autologous matrix-induced chondrogenesis; MSCs, mesenchymal stem cells.

#### 2.3.3 Advantages and future of personalized treatment

Improved surgical accuracy: With the assistance of AI and surgical robots, the damaged site can be accurately located and repaired, reducing damage to surrounding tissues.

Reduced complications: Intelligent treatment can reduce the surgical risks and the occurrence of complications such as postoperative infection and bleeding.

Reduced rehabilitation time: Owing to the small amount of surgical trauma and high accuracy, the rehabilitation time is correspondingly shortened.

**Figure 3:**
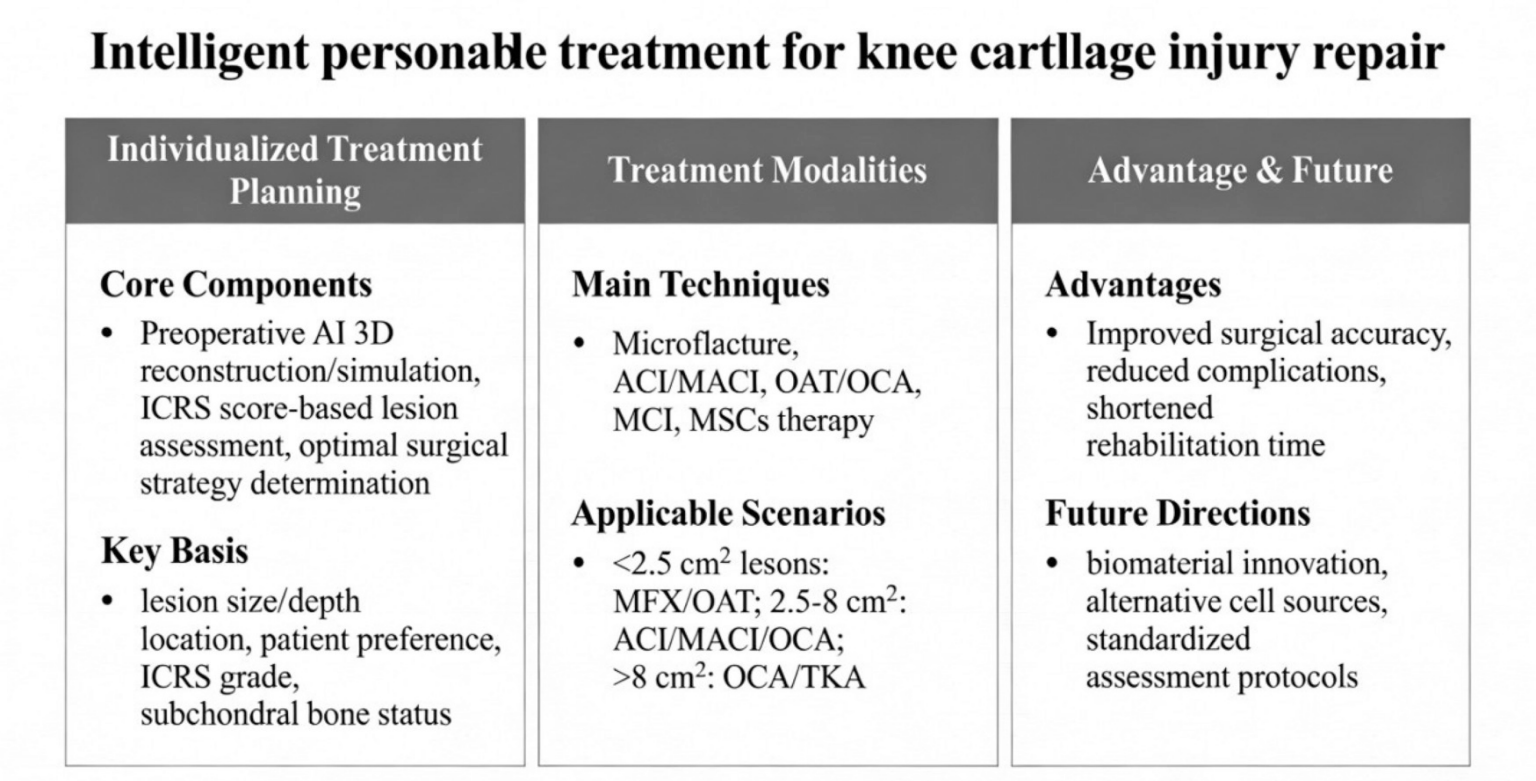
Overview of intelligent personalized treatment for knee cartilage injury repair. AI, artificial intelligence; ICRS, International Cartilage Repair Society; MFX, microfracture; OAT, osteochondral autograft transfer; ACI, autologous chondrocyte implantation; OCA, osteochondral allografts; MCI, minced cartilage implantation; TKA, total knee arthroplasty; MACI, matrix-assisted chondrocyte implantation; AMIC, autologous matrix-induced chondrogenesis; MSCs, mesenchymal stem cells.

The treatment plan for knee cartilage injury should be individualised according to the patient characteristics and the specific circumstances of the injury (26). However, further studies are required to refine the optimal indications for each treatment strategy and standardise the assessment protocol. Future studies should explore the development of novel biomaterials and alternative cell sources to improve cell-based knee cartilage repair therapies (see Figure 3).

Limitations of this review include its narrative (non-systematic) methodology, which may introduce selection bias in the literature reviewed, and the absence of formal quality assessment of included studies. However, where available, we have prioritized evidence from systematic reviews, meta-analyses, and randomized controlled trials in our synthesis.

## 3 Conclusion

The integration of intelligent technologies represents a transformative frontier in the management of knee cartilage injuries, moving the field towards a more precise, personalized, and patient-centric model. Beyond the operating room, intelligent rehabilitation equipment and remote monitoring systems are redefining postoperative recovery. By providing objective, real-time feedback on range of motion, gait, and exercise compliance, these tools empower patients and clinicians alike, facilitating earlier mobilization, improved adherence to rehabilitation protocols, and timely intervention for complications. This continuum of intelligent care, from digital planning to data-guided recovery, holds the promise of not only improving short-term surgical success but also enhancing long-term functional outcomes and quality of life. The characteristics included in the study are shown in Table 3.

**Table 3:** Characteristics of key studies included in this review.

Study	Year	Study design	Technology/Topic	Sample size	Key findings	Evidence level
Moglia et al. (6)	2023	Prospective cohort	Holoknee mixed reality for knee osteotomy	20 participants	Improved task completion time in second trial; positive impact on surgical training	Level II
Luan et al. (3)	2023	Systematic review & meta-analysis	Navigation systems in TKA	28 studies, 2,847 patients	ABN improved alignment vs. CONI; shorter procedure time vs. CN	Level I
Lei et al. (4)	2022	Network meta-analysis	Navigation, robotics, PSI in TKA	38 studies, 4,213 patients	Navigation/robotics reduced misalignment; navigation improved mid/long-term KSS scores	Level I
Mennella et al. (8)	2023	Validation study	Deep learning rehabilitation monitoring	45 patients, 1,080 exercises	ROM accuracy: 89%; compensatory pattern accuracy: 98%	Level III
Shim et al. (9)	2023	RCT	Digital healthcare system post-TKA	46 patients	AR-based rehabilitation effective alternative to conventional rehab	Level II
Neumann-Langen et al. (11)	2023	Prospective cohort	Motion sensor + smartphone app post-TKA	50 patients	Improved QOL (*p* < 0.001), reduced pain (*p* < 0.01) and depression (*p* < 0.01)	Level II
Mehta et al. (12)	2020	RCT	Remote monitoring post-arthroplasty	602 patients	Significantly reduced rehospitalization rate	Level II
Zhao et al. (14)	2024	RCT	Smartphone-based remote rehab post-TKA	80 patients	Effective alternative to face-to-face rehabilitation	Level II
Lippi et al. (15)	2024	Prospective cohort	Step-App® telemonitoring post-arthroplasty	50 patients	Validated tool for remote monitoring	Level II
Orth et al. (19)	2020	Systematic review	Microfracture for cartilage repair	28 studies, 2,052 patients	Failure rates: 11%–27% at 5 years, 6%–32% at 10 years	Level I
Na et al. (20)	2019	Systematic review	ACI vs. microfracture	7 studies, 669 patients	ACI superior to microfracture at 5-year follow-up	Level I
Frodl et al. (22)	2022	Systematic review & meta-analysis	Minced cartilage implantation	10 studies, 368 patients	Comparable short-term improvement to standard techniques	Level I
Razak et al. (24)	2023	Systematic review	MSC implantation for knee OA	16 studies, 558 patients	Short-term clinical improvement; satisfactory cartilage restoration	Level I

TKA, total knee arthroplasty; ABN, accelerometer-based navigation; CONI, conventional instruments; CN, computer navigation; PSI, patient-specific instruments; ROM, range of motion; RCT, randomized controlled trial; QOL, quality of life; ACI, autologous chondrocyte implantation; MSC, mesenchymal stem cells; OA, osteoarthritis; Level I, systematic review/meta-analysis or RCT; Level II, prospective cohort; Level III, validation study.

To realize the full potential of intelligent treatment strategies, several research priorities should guide future investigations. First, prospective randomized controlled trials comparing intelligent vs. conventional care pathways are urgently needed, with standardized outcome measures including not only clinical and patient-reported outcomes but also cost-utility analyses to inform healthcare policy and reimbursement decisions. Such trials should be designed with sufficient follow-up (minimum 5 years) to assess durability of benefits. Second, the development of interoperable data platforms represents a critical infrastructure priority. These platforms should enable seamless integration of preoperative imaging data, intraoperative navigation logs, and postoperative rehabilitation metrics, creating a comprehensive digital patient record that transcends individual care episodes. Such integration would facilitate the “closed-loop” learning healthcare system where surgical outcomes data inform refinements to preoperative planning algorithms, and postoperative recovery trajectories guide personalized rehabilitation protocols. Third, research should explore the optimal human-technology interface, investigating how to present complex data to clinicians in actionable formats without increasing cognitive load, and how to design patient-facing applications that maximize engagement and adherence while minimizing burden. Finally, implementation science research is needed to identify barriers and facilitators to adoption across diverse healthcare settings, ensuring that the benefits of intelligent technologies reach all patient populations equitably.
